# Plasmadiafiltration ameliorating gut mucosal barrier dysfunction and improving survival in porcine sepsis models

**DOI:** 10.1186/s40635-016-0105-2

**Published:** 2016-09-29

**Authors:** Ming Xin Li, Jun Feng Liu, Jian Da Lu, Ying Zhu, Ding Wei Kuang, Jian Bing Xiang, Peng Sun, Wei Wang, Jun Xue, Yong Gu, Chuan Ming Hao

**Affiliations:** 1 Department of Nephrology, Huashan Hospital, Fudan University, 12 Middle Wulumuqi Road, Shanghai, 200040 China; 2 Department of General Surgery, Huashan Hospital, Fudan University, 12 Middle Wulumuqi Road, Shanghai, 200040 China; 3 Department of Critical Care Medicine, Huashan Hospital, Fudan University, 12 Middle Wulumuqi Road, Shanghai, 200040 China

**Keywords:** Cecal ligation perforation (CLP), Sepsis, Plasmadiafiltration, Inflammatory mediators, Intestinal mucosal barrier

## Abstract

**Background:**

The object of this study is to explore whether the plasmadiafiltration (PDF) is more effective in improving the intestinal mucosal barrier function by removing more key large molecular inflammatory mediators and then prolonging the survival time.

**Methods:**

Totally, 24 porcine sepsis models induced by cecal ligation and puncture (CLP) operation were randomly divided into three groups: PDF group, high-volume hemofiltration (HVHF) group, and control group, and received 8 h treatment, respectively. The expression of ZO-1 and occludin in intestinal mucosal epithelial cells were detected by immunohistochemistry, and apoptotic protein caspase-3-positive lymphocytes were signed in mesenteric lymph nodes by TUNEL staining. The hemodynamic parameters were measured by invasive cavity detection. The tumor necrosis factor alpha (TNFα) and high-mobility group protein 1 (HMGB1) were tested by ELISA method. And then, the survival curves with all-cause death were compared with three groups.

**Results:**

PDF led to a superior reversal of sepsis-related hemodynamic impairment and serum biochemistry abnormalities and resulted in longer survival time compared with HVHF and control (*p* < 0.01). Definitive protection from excessive TNF-α and HMGB1 response were only achieved by PDF. A more regular distribution pattern of ZO-1 and occludin along the epithelium was found in PDF animals (*p* < 0.01). The presence of apoptotic lymphocytes was significantly reduced in the PDF animals (*p* < 0.01).

**Conclusions:**

PDF can effectively eliminate more pivotal inflammatory mediators of TNFα and HMGB1 and reduce the inflammation damage of the intestinal mucosal barrier and apoptosis of lymphocyte then improve the circulation function and prolong the survival time.

## Background

During sepsis, the most frequent complication within the gastrointestinal tract is mucosal barrier dysfunction. Gut barrier lesion plays an important role in the pathophysiology of sepsis by promoting bacterial translocation [1, 2], which leads to secondary infection and multiple organ failure. The intestinal mucosal barrier consists of mechanical and immunological barrier. Unfortunately, the cascade of inflammation directly injuries intestinal epithelial tight junction permeability [3], particularly for tumor necrosis factor alpha (TNFα) and high-mobility group protein 1 (HMGB1) [4], by impairing the function of mitochondria [5, 6]. And meanwhile cytokine storm uptakes apoptosis of lymphocytes and intestinal epithelium cells via caspase active [7, 8]. Accordingly, epithelial dysfunction may be a common final pathway contributing to organ dysfunction in sepsis and other forms of critical illness. It plays a key role in the prognosis of sepsis and it should become the focus of the sepsis treatment.

The overall therapies and concept of blood purification have evolved toward non-specifically removing a broad spectrum of plasma inflammatory mediators and also attenuating the tissue overwhelming systemic expression of pro- and anti-inflammatory mediators [9, 10]. Rebuilding of immune homeostasis is thought to be able to improve outcomes and survival. Plasmadiafiltration (PDF), which used high cut-off hemofilter and diluted plasma as replacement fluid, represents new logical strategy to significantly increase middle- and high-molecule-weight mediator removal with low substitution flow rate. Compared to coupled plasma filtration and absorption or other plasmapheresis, PDF possesses better restoration of homeostasis and similar efficient cytokine clearance with simpler therapeutic modality. And meanwhile, a little fresh iced plasma complement essential coagulation factors and immunoglobulin that benefits sepsis recovery.

Our previous in vitro study [11] had demonstrated the safety and effectiveness of PDF for removing a sufficiently wide of inflammatory mediators and small molecular toxins from circulation, and it provided acceptable albumin depletion by a little plasma supplement.

This in vivo study was to explore whether PDF was more effective improving the intestinal mucosal barrier function by removing more key large molecular inflammatory mediators and then prolonging the survival time than that of classical hemofiltration.

## Methods

### Anesthesia and surgical preparation

The Animal Care Committee of the Fudan University approved the study. Twenty-four clean grade minipigs (body weight [BW] 36 ± 1.3 kg, aged 80 ± 1.2 days) were premedicated intramuscularly with ketamine (10–15 mg/kg) and diazepam 10 mg. Anesthesia ventilator (Matrx Model3000, USA) maintained with inhalation of mixture 1–5 % isoflurane and oxygen by an endotracheal intubation. A catheter was inserted in the left carotid artery for blood pressure monitoring and blood sampling. Two 7 French Swan-Ganz flow-directed pacing catheters (131F7, 1.5 ml CAP, Edwards, USA) were introduced aseptically via two 8 Fr. introducers (Exacta Percutaneous Sheath Inducer) into the right proximal and distal jugular veins, under continuous pressure monitoring, their tips were advanced into the pulmonary artery and right atrium, respectively. During anesthesia, the animals received continuous infusions with isotonic sodium chloride at 5 ml/kg/h. Baseline data were collected.

### CLP models

CLP operation was performed with laparoscopic surgery (Olympus, Japan). Four incisions of 0.5–1.0 cm were made on the insufflated abdomen with carbon dioxide gas and trocars were placed through these incisions, then laparoscope and other instruments were introduced into the abdomen through these trocars. The cecum was identified and exposed and a specified 6 cm of the distal end of the cecum was ligated. Next, one incision of 1 cm at the free end of the cecum was made. The cecum was then gently compressed to extrude amount of cecal content. At last the instruments were pulled out and the abdominal skin incisions were closed. After resuscitation the porcines of CLP were backed animal room with free diet.

### Experiment protocol

After 24 h of CLP operation, the experimental animals were induced with forward mentioned inhalation anesthesia and allocated into PDF, high-volume hemofiltration (HVHF) and control group in accordance with random digital generated by Stata 10.0 software (Stata Co. Ltd, USA). And the animals were then recannulated to begin the experimental period. The control group treated with hemoperfusion through an empty column with the same blood flow rate and anticoagulation method as that of the PDF and HVHF. All the animals received a continuous infusion of isotonic sodium chloride. The infusion rate was adjusted according to the clinical situation and to reach target central venous pressure of 5–10 cmH_2_O. After a maximal therapeutic and observation period of 32 h, animals were lethally injected with potassium chloride.

### Plasmadiafiltration

PDF therapies were delivered by the KM8900 machine (Quarary, Japan), and the EVAL membrane hollow fiber dialyzers were used, which had high cut-off of 60–70 KD (Evacure EC-2A, inside diameter 175 μm, wall thickness 40 μm, and effective surface area 1.0 m^2^, Sichuan Chemical Industry, Japan). Blood flow rate was set as 2.5 ml/kg/min throughout the whole experiment while the replacing fluid that was consisted of commercially available substitution and plasma at the ratio of 3:1 was infused in a post-dilution mode at the desired rate of 4 ml/kg/h to maintain a zero fluid balance. Dialysate flow rate was set at 1000 ml/h consistently and net ultra filtration rate adjusted according to central venous pressure. For anticoagulation, porcines were given a loading dose of 6250 U heparin, followed by a continuous infusion of 625 U/h.

### High volume hemofiltration

Zero-balanced hemofiltration was performed in a predilution mode with rate of 60 ml/kg/h using a polysulfone membrane (F60 HPS, an area of 1.3 m^2^, Fresenius, Germany) connected to a Diapact machine (B.Braun, Germany). Blood flow rate and anticoagulation method were designed as same as PDF treatment.

### Vital signs assessment, biochemistry data, and cytokine measurement

Internal temperature, heart rate, respiratory rate, arterial blood pressure, blood oxygen saturation, cardiac output, pulmonary arterial wedge pressure, and central venous pressure were observed by multichannel physiological monitor (Agilent^®^, Polymount GCX Corporation, USA) with different transducer connected to left carotid artery catheter and right jugular venous Swan-Ganz catheter respectively at 0, 24 h, and then every 15 min after treatment. And urine volume per hour was documented with catheterization.

Furthermore, blood sample were obtained for determination of leucocyte counts, serum sugar, creatinine, lactate, and blood gases at points 0 and 24 h and then every 2 h after treatment with COULTER LH750 (Beckman Coulter, USA) and ISTAT® Handheld (Abbott Point of Care, USA).

Serum samples were gathered at the same time point and stored in −70 °C for later cytokines assay. The concentrations of HMGB1 and TNF-α trimer were detected by specific enzyme-linked immunosorbent assay (ELISA) kits, following the protocols of the manufacture (R&D Systems Co., Minneapolis, MN).

### Immunohistochemistry of ZO-1 and occludin

Immediately after death of CLP porcine, the 5 cm ileum section was obtained, and randomly sampled tissue blocks of approximately 50 × 50 × 50 μm were fixed over night in 10 % (wt/vol) PBS-buffered formaldehyde, following the protocols of the former paper described [12]. The positive areas were captured by Nikon System (Nikon NIS-Elements, Nikon Eclipse 50i H550S, DS-FiI, Nikon, Japan) and then immunochemistry index referred to the ratio of mean optical density/area was calculated with photo analysis software Image pro® plus 6.0 (Media Cybernetics, USA). And all immunohistology data were quantitatively analyzed by Shanghai Showbio Biotechnology Company, as a blinded independent third-party testing agency.

### TUNEL assay for caspase-3-positive lymphocytes

Mesenteric lymph nodes were obtained pre-CLP and at the moment of death, respectively, and were fixed as forward mentioned method. The terminal deoxynucleotidyl transferase mediated dUTP-biotin nick end labeling (TUNEL) assay was used to observe apoptotic lymphocytes in the mesenteric lymph node, following the protocols of the former paper described [12]. Brownish-yellow stained lymphocytes were an indication of positive cells. The apoptotic lymphocytes were identified and counted.

### Statistical methods

All numerical data were expressed as mean ± standard error. Normal distributed data with homogeneity or heterogeneity of variance was compared with Student’s *t* tests or Welch’s *t* test. And skewed distribution data was used Wilcoxon rank test. Multiple linear regressions were used to determine an association between cytokines and hemodynamic parameters. Survival times were calculated by Kaplan-Meier analysis and compared by the log-rank test. All analyses were performed by Stata software package 10.0 (StataCorp LP, College Station, TX, USA). A *p* < 0.05 was considered to be of significant difference.

## Results

### Clinic data and laboratory parameters

All animals possessed the similar physiological attributes at baseline or after 24 h CLP operation between three groups (Table 1). And meanwhile, all CLP porcines conformed to sepsis shock diagnostic standard after 24 h, according to the updated sepsis 3.0 criteria [13], the occurrence of sepsis shock was assumed SOFA score ≥2 (sequential organ function assessment (SOFA)) associated with at least two of the following signs: systolic blood pressure ≤100 mmHg and serum lactate ≥2 mmol/l.

**Table 1:** Comparison of hemodynamics and blood variables in three groups

Variable	Group	Time (hour)					
		Baseline	24	26	28	30	32
PaO^2^ (mmHg)	PDF	108.9 ± 20.1	66.5 ± 18.5a^a^	74.50 ± 5.79^b^	84.67 ± 6.95^b^	93.00 ± 5.18	101.0 ± 5.33
	HVHF	106.9 ± 23.1	65.8 ± 19.8a^a^	68.83 ± 3.19	74.17 ± 3.26	81.83 ± 1.94	89.50 ± 4.76
	Con.	110.2 ± 21.9	68.0 ± 16.9a^a^	58.50 ± 2.43	54.67 ± 2.73	51.00 ± 2.00	
MAP	PDF	77.7 ± 2.07	54.50 ± 1.87^a^	56.33 ± 1.63	58.33 ± 1.63 ^b^	60.50 ± 1.87	62.50 ± 1.87
	HVHF	79.2 ± 1.93	52.83 ± 1.72^a^	51.83 ± 1.33	50.33 ± 0.82	50.67 ± 1.75	52.53 ± 1.87
	Con.	76.9 ± 1.89	52.50 ± 1.87^a^	50.00 ± 2.00	47.33 ± 2.25	44.83 ± 1.84	
PAWP	PDF	11.35 ± 2.85	22.00 ± 3.74^a^	19.67 ± 2.58	17.67 ± 1.97^b^	16.17 ± 1.47	14.00 ± 1.79
	HVHF	11.48 ± 2.32	23.00 ± 3.74^a^	22.33 ± 3.27	21.33 ± 3.20	20.50 ± 3.08	20.17 ± 3.43
	Con.	12.01 ± 3.07	20.50 ± 1.87^a^	22.33 ± 3.01	24.00 ± 3.63	25.83 ± 3.76	
CO	PDF	2.29 ± 0.56	2.12 ± 0.25^a^	2.10 ± 0.26	2.10 ± 0.28 ^b^	2.18 ± 0.27	2.19 ± 0.26
	HVHF	2.33 ± 0.74	2.19 ± 0.27^a^	2.09 ± 0.24	1.98 ± 0.22	1.82 ± 0.16	1.71 ± 0.17
	Con.	2.31 ± 0.42	2.18 ± 0.28^a^	1.83 ± 0.28	1.53 ± 0.15	1.29 ± 0.10	
Urine flow (ml/h)	PDF	95.4 ± 0.87	18.33 ± 1.63^a^	20.00 ± 1.41	23.00 ± 2.19 ^b^	26.17 ± 2.40	29.33 ± 2.34
	HVHF	97.6 ± 0.90	20.00 ± 2.61^a^	19.33 ± 2.16	18.67 ± 2.42	18.00 ± 2.28	17.50 ± 2.88
	Con.	96.5 ± 0.73	20.67 ± 2.42^a^	15.67 ± 3.78	10.33 ± 3.89	6.17 ± 4.54	
WBC ( × 10^9^/L)	PDF	13.8 ± 4.3	26.8 ± 5.3a^a^	25.8 ± 4.9	23.6 ± 5.1	22.7 ± 4.5	20.8 ± 5.5
	HVHF	12.9 ± 5.6	27.7 ± 5.9a^a^	26.2 ± 6.1	24.5 ± 3.9	23.2 ± 6.8	21.9 ± 7.5
	Con.	15.1 ± 6.2	23.4 ± 4.2a^a^	25.8 ± 7.3	24.4 ± 4.2	26.3 ± 4.4	
Scr (μmol /l)	PDF	83.5 ± 15.3	143.1 ± 76.4^a^	131.2 ± 56.3	127.4 ± 26.8	109.7 ± 21.4	97.62 ± 23.5
	HVHF	85.8 ± 12.5	134.7 ± 86.3^a^	149.5 ± 66.7	142.3 ± 45.2	138.3 ± 33.4	120.5 ± 32.8
	Con.	81.5 ± 10.9	140.3 ± 80.4^a^	155.9 ± 67.3	178.2 ± 55.4	195.8 ± 32.1	
LAC (mmol/l)	PDF	1.47 ± 0.42	4.28 ± 1.40^a^	3.26 ± 1.12	2.21 ± 1.08^b^	1.97 ± 0.76	1.74 ± 0.45
	HVHF	1.58 ± 0.35	4.32 ± 1.18^a^	3.47 ± 1.16	3.01 ± 0.97	2.14 ± 0.65	1.88 ± 0.56
	Con.	1.35 ± 0.52	3.94 ± 1.32^a^	4.97 ± 1.54	5.87 ± 1.17	7.45 ± 0.49	
Survival rate	PDF	8/8	8/8	8/8	8/8	7/8	4/8
	HVHF	8/8	8/8	6/8	4/8	2/8	2/8
	Con.	8/8	8/8	3/8	2/8	2/8	0/8

*PDF* plasmadiafiltration, *HVHF* high-volume hemofiltration, *Con*. control, *PaO* ^*2*^ partial pressure of blood oxygen, *MAP* mean arterial pressure, *PAWP* pulmonary artery wedge pressure, *CO* cardiac output, *WBC* white blood cell, *Scr* serum creatinine, *LAC* lactate

^a^ *p* < 0.01 vs. the respective value at baseline

^b^ *p* < 0.01 vs. the HVHF or control

**Fig. 1:**
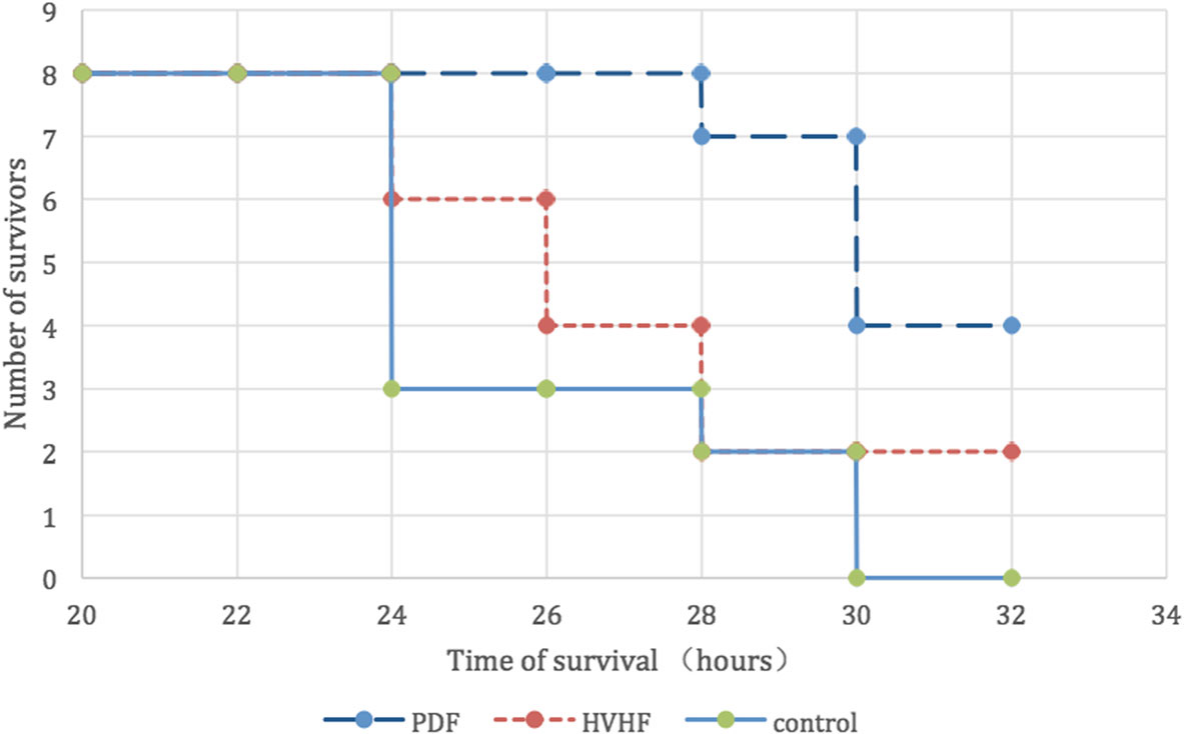
Effects of extracorporeal blood purification on survival of cecal ligation puncture (CLP) porcine. Survival time (hours) was observed from the start of CLP. *p* < 0.01, PDF vs. HVHF or control group, respectively

After the onset of sepsis shock animals exhibited macrocirculatory derangement characterized by decreases in mean arterial pressure (MAP) and cardiac output (CO), and pulmonary disorders indicated by increase in pulmonary artery wedge pressure (PAWP) and decrease in partial pressure of blood oxygen (PaO_2_), (Table 1). PDF led to a distinctly superior reversal of sepsis-related hemodynamic impairment compared with HVHF and control. And meanwhile, the values of serum lactate, creatinine, and leucocyte were elevated after 24 h postinduction of CLP in all groups and much more normalized during the course of disease in PDF than in HVHF and control, only except leucocyte. This resulted in a definitive prevention of sepsis shock by PDF in a total of four porcines, two of which belonged to HVHF and no animals from control survived (see Fig. 1).

### Changes of cytokines

**Fig. 2:**
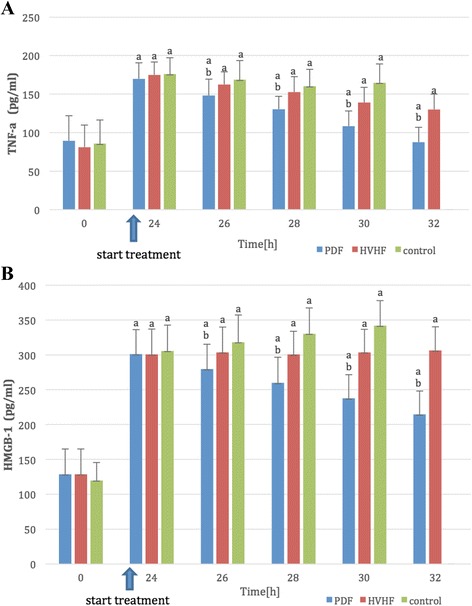
Plasma tumor necrosis factor (TNF-α) (**a**) and high mobility group protein 1 (HMGB1) (**b**) levels obtained at different points of time. Definitive protection from overwhelming TNF-α and HMGB1 response were only achieved by plasmadiafiltration (PDF). Attenuation of cytokines was most effective in animals undergoing PDF. (*a*) *p* < 0.01 vs. 0 h; (*b*) *p* < 0.01 vs. HVHF or control, respectively

Differences in these circulating plasma cytokine concentrations for septic shock porcines therapy with PDF, HVHF, or control were shown in Fig. 2. Baseline or values of 24 h after CLP were not different among the three groups for any cytokine. However, CLP resulted in an increase of TNF-α and HMGB1 level. PDF more efficiently attenuated the TNF-α and HMGB1 level in serum (Fig. 2). And meanwhile, by multiple linear regressions analysis, only serum TNF-α level was significantly negative associated with MAP and CO, respectively (*p* < 0.05).

### Effects of sepsis on gut tight conjunction

**Fig. 3:**
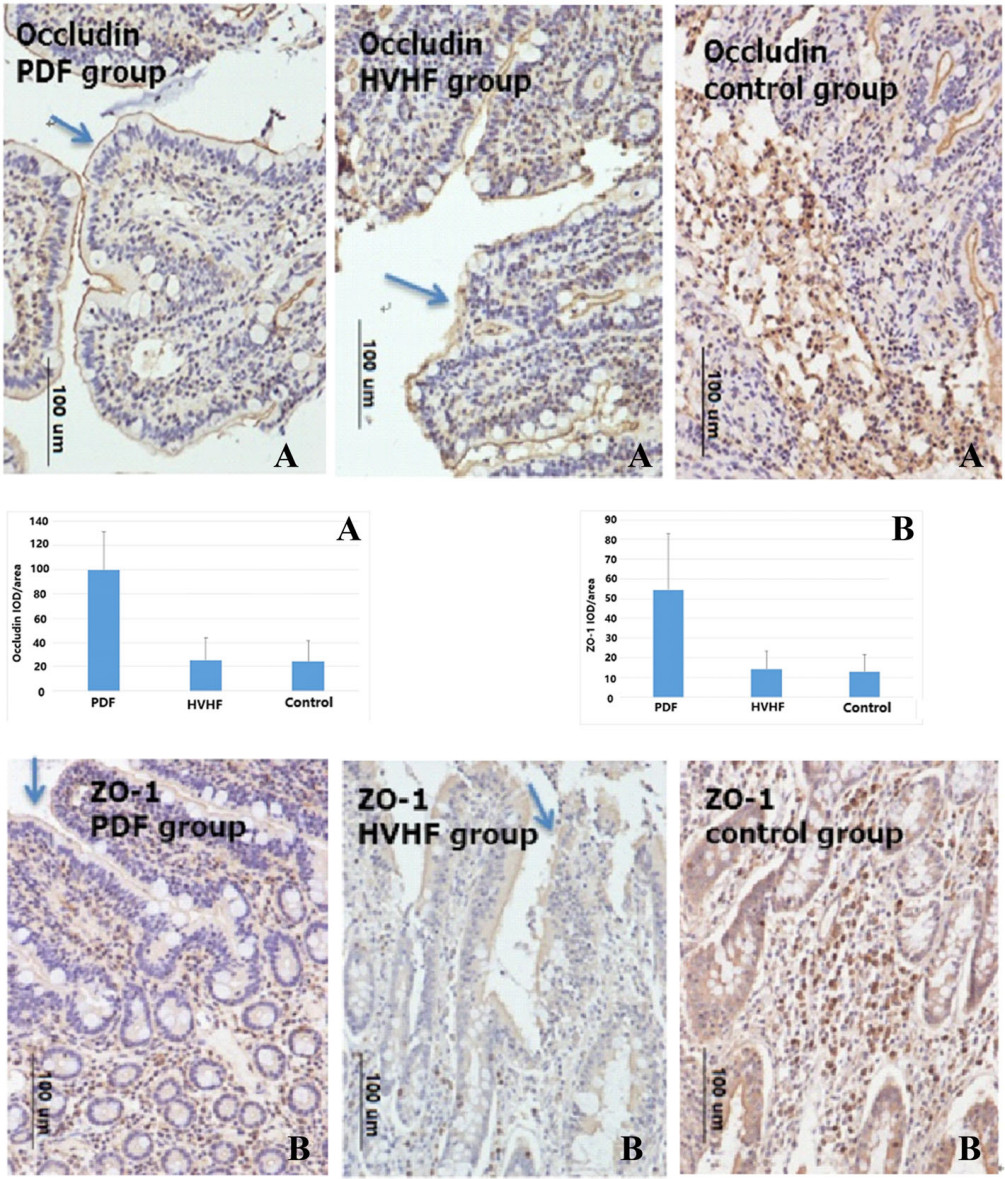
Immunohistochemical localization of zonula occludens (ZO)-1 (**b**) and occludin (**a**) in the ileum tissue from cecal ligation puncture (CLP) porcines. A more regular distribution pattern of ZO-1 and occludin (see *arrow*) along the epithelium was found in PDF animals. Contrastingly, a significant disruption of immunosignal for them was observed along the epithelium in HVHF (see *arrow*) and control group, particularly for control animals; almost all-normal construction disappeared and infiltrated with a lot of inflammatory cells. *p* < 0. 01 PDF group vs. HVHF group, or control group, respectively. *IOD* immunochemistry optical density

ZO-1 and occludin were measured by immunohistochemistry in ileum sections. Results indicate that, in the ileum tissues collected from CLP porcines, ZO-1 and occludin were comparatively continuously distributed along the epithelium in PDF group. In contrast, a significant disruption of immunosignal for them was observed along the epithelium in HVHF and control group, particularly for control animals, almost all-normal construction disappeared and infiltrated with a lot of inflammatory cells. (Fig. 3a, b)

### Numbers of apoptotic lymphocytes in the mesenteric lymph nodes

**Fig. 4:**
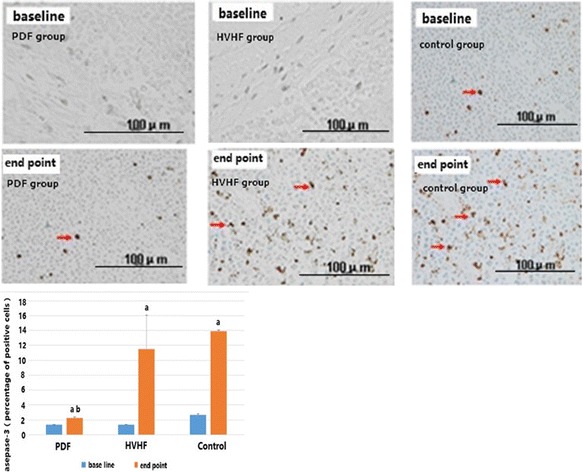
Effects of cecal ligation puncture (CLP) induced sepsis on apoptosis of lymphocytes. Almost no apoptotic cells were detected in mesenteric lymph nodes at baseline. At the endpoint of CLP induced sepsis shock, mesenteric lymph nodes from CLP animals demonstrated a marked appearance of *dark brown* apoptotic cells and intercellular apoptotic fragments (see *arrow*). The presence of apoptotic cells or fragments was significantly reduced in the PDF animals. (*a*) *p* < 0.01 vs. baseline; (*b*) *p* < 0.01

Almost no apoptotic lymphocyte cells were detected in mesenteric lymph nodes at baseline (Fig. 4). At the endpoint of CLP induced sepsis shock, mesenteric lymph nodes from CLP animals demonstrated a marked appearance of dark brown apoptotic cells and intercellular apoptotic fragments (see arrow in Fig. 4). The presence of apoptotic cells or fragments was significantly reduced in the PDF animals (Fig. 4).

## Discussion

Polymicrobial sepsis induced by cecal ligation and puncture (CLP) is the most frequently used model because it closely mimics the progression and attributes of human sepsis [14]. In addition, the higher the ligation and the bigger the incision size, the higher the mortality. Actually, most of sepsis-induced patients usually suffer from multiple organs dysfunction and sepsis shock in the clinic practice. Here, CLP with higher ligation and bigger incision successfully induced serious illness model that was full compliance with sepsis shock diagnostic standard after 24 h, according to the updated sepsis 3.0 criteria [13], and which completely recreated human sepsis progression with similar hemodynamic and metabolic phases and the presence of both hyper- and hypoinflammatory phases, and also with prolonged and lower elevation of cytokine release, as in humans.

Recent technological progress has increased the number of techniques available for blood purification and their performance [15–17]. Unfortunately, recent a series of large multiple centers randomized controlled trials demonstrate that neither the different technique [18–20] nor the treatment dosage [21–24] had an impact on patient survival. Otherwise, PDF, the newer hybrid technique, which combines the hemodynamic and homeostasis stability of continuous renal replacement therapy with a more efficient depletion of broad spectrum of plasma inflammatory mediators and toxins similar to plasmapheresis, has been introduced as a new cost-effective simple approach to the treatment of acute kidney injury (AKI) in the sepsis shock. Our previous in vitro study [11] had demonstrated the safety and effectiveness of PDF for removing a sufficiently wide of inflammatory mediators and hypercatabolic toxins from circulation, and it provided acceptable albumin depletion by a little fresh iced plasma supplement that also benefited recovery of coagulation and immunity.

Delayed therapy was performed in this experiment protocol differentiated to previous animal research in order to preferably simulate the clinic practice. And the choice of 8 h treatment was supported from the results that daily hemodialysis or hemofiltration presented excellent detoxification with cardiovascular tolerability and similar outcomes [25–27], and meanwhile ameliorated anticoagulant dosage and disturbance of antibiotic concentration compared with continuous blood purification [28]. As expected result, PDF demonstrated significantly more effective prevention of sepsis-associated hemodynamic deterioration than HVHF and control. And meanwhile, the values of serum lactate, creatinine, and leucocyte were elevated after 24 h postinduction of CLP in all groups and much more normalized during the course of disease in PDF than in HVHF and control, only except leucocyte. This resulted in a definitive prevention of sepsis shock by PDF in a total of four porcines, two of which belonged to HVHF and no animals from control survived.

In our study, favorable clinical outcomes that were convincible elucidated that PDF with high cut-off (HCO) membrane was more efficient in regard to cytokine clearances, improved injury of gut tight junction and ameliorated apoptosis of lymphocytes. In animal models of sepsis, HCO membranes improve hemodynamics and prolong survival [29]. In patients with sepsis-related acute kidney injury, Morgera and colleagues [30] reported a significant higher clearance of cytokines and a reduction in vasopressor requirements with the use of HCO hemofiltration. In another randomized research, HCO hemofiltration restored the monocyte proliferation capacity of septic patients, probably by eliminating immunomodulatory mediators [31]. As is known, connective removal of a solute for high cut-off membrane depends on trans membrane pressure, which is conditioned by the rate of substitution, ultrafiltration, and dialysate. In order to achieve an acceptable balance between high cytokine and low albumin clearances, based on our former in vitro research result [11], we designed optimal combination of replacement, ultrafiltration, and dialysate as PDF protocol description. Although lessening a little bit inflammation factor clearance, it ensured enough cytokine removal and minimum albumin lost.

In inflammation, overproduction of TNF-α is pivotal in the induction of inflammatory genes, cell death, and in the recruitment and activation of immune cells [32]. In addition, it has been demonstrated that TNF-α plays a role in the control of epithelial permeability [33–35]. Moreover, TNF-α at higher concentrations leads to downregulation of ZO-1 protein expression and disturbance in junction localization of ZO-1 protein and functional opening of TJ barrier [12, 35, 36]. HMGB1, a potent late proinflammatory mediator in sepsis, also increased permeability of cultured epithelial monolayers in vitro and murine ileal mucosa in vivo [4]. Furthermore, a serious mechanism researches reveal inhibiting gut epithelial apoptosis by overexpression of Bcl-2 was associated with a survival advantage in pseudomonas pneumonia-induced sepsis. This result suggested that intestinal epithelial apoptosis played a role in sepsis-related mortality [37]. And Dr. Julian’s findings indicated that intestinal epithelium was more susceptible to mitochondrial damage and dysfunction than the lung epithelium in the context of sepsis [5]. Sepsis-induced inflammatory cells infiltration accompanied with cytokine releasing, epithelial apoptosis, and mitochondria dysfunction all involved in injury of gut tight junction [38, 39]. Therefore, intestinal failure is common in patients with septic shock, with dysfunction of the gut often manifesting as both a cause and consequence of their critical illness [40].

Actually, CLP resulted in an increase of TNF-α and HMGB1 level, a marked appearance of dark brown apoptotic lymphocyte cells, and a significant disruption of immunosignal for ZO-1 and occludin along the epithelium. It is very gratifying to see that PDF effectively attenuated the TNF-α and HMGB1 levels in serum (Fig. 2) and also improved apoptosis of lymphocyte and sepsis-related gut barrier injury.

## Conclusions

PDF can effectively eliminate more large pivotal inflammatory mediators of TNFα and HMGB1 and improve the sepsis-related gut barrier dysfunction and apoptosis of lymphocyte, and meanwhile, it benefits the circulation function and prolong the survival time. In the future, large multicenter randomized controlled trials will further appraise the power of PDF therapies to reduce mortality or organ failure, rather than only concerning on cytokine clearance or transient improvement in physiologic variables.

## Abbreviations

AKI: Acute kidney injury
CLP: Cecal ligation and puncture
CO: Cardiac output
Con.: Control
ELISA: Enzyme-linked immunosorbent assay
HCO: High cut-off
HMGB1: High-mobility group protein 1
HVHF: High-volume hemofiltration
LAC: Lactate
MAP: Mean arterial pressure
PaO_2_: Partial pressure of blood oxygen
PAWP: Pulmonary artery wedge pressure
PDF: Plasmadiafiltration
Scr: Serum creatinine
SOFA: Sequential organ function assessment
TNFα: Tumor necrosis factor alpha
TUNEL: Terminal deoxynucleotidyl transferase mediated dUTP-biotin nick end labeling
WBC: White blood cell
